# Identifying Temporally Regulated Root Nodulation Biomarkers Using Time Series Gene Co-Expression Network Analysis

**DOI:** 10.3389/fpls.2019.01409

**Published:** 2019-10-31

**Authors:** William L. Poehlman, Elise L. Schnabel, Suchitra A. Chavan, Julia A. Frugoli, Frank Alex Feltus

**Affiliations:** Department of Genetics and Biochemistry, Clemson University, Clemson, SC, United States

**Keywords:** root, nodulation, symbiosis, biomarker, network, bioinformatics, ribonucleic acid sequencing, Knowledge Independent Network Construction

## Abstract

Root nodulation results from a symbiotic relationship between a plant host and *Rhizobium* bacteria. Synchronized gene expression patterns over the course of rhizobial infection result in activation of pathways that are unique but overlapping with the highly conserved pathways that enable mycorrhizal symbiosis. We performed RNA sequencing of 30 *Medicago truncatula* root maturation zone samples at five distinct time points. These samples included plants inoculated with *Sinorhizobium medicae* and control plants that did not receive any *Rhizobium*. Following gene expression quantification, we identified 1,758 differentially expressed genes at various time points. We constructed a gene co-expression network (GCN) from the same data and identified link community modules (LCMs) that were comprised entirely of differentially expressed genes at specific time points post-inoculation. One LCM included genes that were up-regulated at 24 h following inoculation, suggesting an activation of allergen family genes and carbohydrate-binding gene products in response to *Rhizobium*. We also identified two LCMs that were comprised entirely of genes that were down regulated at 24 and 48 h post-inoculation. The identity of the genes in these modules suggest that down-regulating specific genes at 24 h may result in decreased jasmonic acid production with an increase in cytokinin production. At 48 h, coordinated down-regulation of a specific set of genes involved in lipid biosynthesis may play a role in nodulation. We show that GCN-LCM analysis is an effective method to preliminarily identify polygenic candidate biomarkers of root nodulation and develop hypotheses for future discovery.

## Introduction

Root nodulation is a symbiotic process in which a plant host allows *Rhizobium* to colonize roots in unique plant organs called nodules. The plant provides carbon to the *Rhizobium* in exchange for ammonium that is produced by atmospheric nitrogen fixation (Suzaki and Kawaguchi, 2014). *Medicago truncatula* is a model plant that produces indeterminate nodules that persistently grow from a meristem (Gage, 2004). In response to inoculation with *Rhizobium* such as *Sinorhizobium medicae*, genetic pathways are activated to initiate and maintain nodule development. Nod factor lipoproteins that are released by the *Rhizobium* interact with receptor-like kinases in the plant, resulting in a spike in calcium oscillations from the nucleus of the cell that activates signaling pathways necessary to produce nodules (Oldroyd, 2013). These signaling pathways result in the production of proteins that allow the *Rhizobium* to enter and colonize the host plant (infection thread formation), and nodule organogenesis ensues from rapid cortical cell division (Long, 2001; Jones et al., 2007).

Many events occur within a few hours of infection in the elongation zone of the root, but trigger later morphological changes, reviewed in Oldroyd and Downie (2008), that are tied to the transcriptomes generated in this work. Root hairs in cells that have ceased to elongate do not respond to rhizobia (Gage, 2004). Upon attachment of the rhizobia to the root hair tips, the root hairs curl tightly and entrap the bacteria in the curl. The plant cell forms a new structure, a tubular infection thread, through which the bacteria enter the plant through cell division. At the same time as infection thread formation, a subset of the inner cortical cells next to the xylem poles is mitotically activated and these cells will eventually form the nodule primordia. We do not know what the signal is that reactivates the cell cycle, although *ENOD40* is required (Charon et al., 1997), but *in situ* analysis of ACC synthase activity in wild type plants and examination of ethylene insensitive *M. truncatula* mutant strongly suggests that this positional information is related to ethylene levels in the cortical cells between the xylem poles (Heidstra et al., 1997; Penmetsa and Cook, 1997; Penmetsa et al., 2008).

As reviewed in Oldroyd and Downie (2008), within the next 24 h, the infection threads cross the outer cortical cells and begins to branch. The outer cortical cells undergo rearrangements reminiscent of phragmoplast formation that allow the infection threads to pass through the cells. But only a subset of initiated infection threads will persist into the inner cortex; the majority are arrested in the outer cortex and the mechanism for this is unknown (Gage, 2004). Meanwhile the inner cortical cells continue to divide, and the concentrations of both cytokinin and auxin change in the cortex and endodermis/pericycle area. Measurements on whole roots show an increase in cytokinin levels (reviewed in Suzaki et al., 2013) and a reduction of auxin transport from the shoot to the root (van Noorden et al., 2006), but this has not been examined in detail at the level of individual cells, except to note that Nod factor affects polar auxin transport only when applied to the elongation zone of the root, where the nodules will form (Suzaki et al, 2012).

By 48 h after inoculation the nodule primordia have begun to organize into a meristematic region and a region with cells that have ceased dividing behind it. In *M. truncatula*, the genes encoding CLE12 and CLE13 peptides are expressed in the meristematic area (Mortier et al., 2010) and are involved in autoregulation, sending a signal to the shoot that nodules are developing (Okamoto et al., 2013). Local changes in auxin transport in the vascular bundle occur where nodules are forming (Mathesius et al. 1998; Huo et al., 2006) similar to what happens when lateral roots initiate, and a subset of auxin transporters (*PIN* genes) are required for nodule development as in lateral root development (Huo et al., 2006).

At 72 h after inoculation, *M. truncatula* will halt the initiation of additional nodule primordia in the elongation zone, presumably in response to an autoregulatory signal (Gage, 2004; Kassaw and Frugoli 2012; Soyano and Kawaguchi, 2014). Under controlled conditions this results in a fixed number of nodules in a small area of the root. Bacteria in infection threads that have entered the outer cortex stop dividing and the threads degrade, auxin transport resumes a normal pattern, and the successful infection threads will begin to release bacteria in symbiosomes into the cells that have ceased to divide behind the meristem (Gage, 2004). The developing nodule is still within the cortex and the vasculature that will feed it has not yet organized, but from this point on the development of the nodule seems to be controlled by signals from the meristem and signals from the bacteria within the symbiosomes.

Temporally coordinated gene expression patterns are necessary to initiate and regulate root nodule formation (Ferguson et al., 2019). Transcriptome profiling has identified genes that are induced upon inoculation with Rhizobium or application of Nod factor. The NIN transcription factor is a master regulator of nodulation, playing roles in nodule organogenesis in cortical and epidermal root cells (Vernie et al., 2015). Other key genes that are induced upon rhizobial infection, formerly termed nodulin genes, have been identified by expression analysis (El Yahyaoui et al., 2004; Larrainzar et al., 2015). While differential gene expression analysis of root transcriptomes has helped to identify such genes, analyzing the whole root tissue is likely diluting the signals from genes that are dynamically involved in nodule organogenesis which occurs in a defined section of the root. For example, CRE1, a cytokinin receptor, is expressed in the root cortex and is associated with young nodule primordia (Lohar et al., 2006). For much of nodule development after the first few hours, the physical process is known from observations via microscopy, but the underlying molecular signals are only known at a gross (whole root) level. This transcriptome analysis focuses on the later physical events.

We reasoned that analyzing the transcriptome of only the portion of the root in which nodules develop could reveal gene expression dynamics that were not detectable from whole-root tissue and so we focused on the area of the root undergoing the morphological changes in response to rhizobia. The root maturation zone is above the meristematic and elongation zones of the root, where the cells stop elongating rapidly and differentiate. It is defined as the part of the root from the first cell with an emerging root hair to the cell with a fully emerged root hair (Ivanov and Dubrovsky, 2013). In M. truncatula and other legumes, this region of the root is also the site of nodule initiation, as only immature emerging root hairs can respond to rhizobia (Gage, 2004). As the root matures and the root hairs mature, the initial region responding initially now becomes unresponsive to rhizobia and moves up farther from the root tip, but nodule development continues to occur in the original cells once initiated (Oldroyd and Downie, 2008; Moreau et al., 2011). Because of the spatial-temporal anatomy of a growing root, identifying sets of genes that are both spatially and temporally regulated during nodulation presents a challenge.

Preliminary experiments in our lab for the proposal that funded this work used an aeroponic system in which all plants grow at the same time in the same media and receive rhizobial inoculation at the same time (**Figure S1**, Materials and Methods). This allowed us to develop a protocol to consistently harvest the inoculated maturation zone over time. We began by microscopically examining a subset of roots (guide roots not being used for the transcriptomics analysis) at the 0 h time point and used a non-destructive marker to mark the top and bottom of the maturation zone. In multiple preliminary trials, this root segment consistently began approximately 1 cm from the root tip and ended 2 cm further up the root. These guide roots were then reloaded to continue growing throughout the experiment. At each time point, the guide roots could be used at each time point for determining the location of the initial zone on the other roots, and the 2 cm length of the zone did not change because the maturation zone no longer elongates.

Our sampling strategy for time evolved from the experiments by Larrainzar et al. (2015) which use the same aeroponic apparatus. We adopted their time course, but because we are focused on the transcriptomic signature of morphological events, we eliminated the 3 and 6 h time points used in their analysis and started with the first time point tied to a physical event—a 12 h time point at which root hair deformation can be observed. To eliminate circadian variation past the initial 12 h time point, we removed the 36 h time point and added a 72 h time point. This was based on our observation that in this aeroponic system at 72 h, nodules began to emerge from the root. Thus except for the 12 h time point, we have minimized circadian variation from our analysis. In this work we also deliberately did not examine differential expression between time points in uninoculated roots. While such an analysis would identify genes involved in root development that could also influence nodulation, our initial goal was to find genes with differential expression only in response to rhizobia.

Gene co-expression network (GCN) analysis is a method that can be applied to elucidate complex gene expression patterns over the time course of root nodulation. A GCN is a graph in which nodes represent genes and edges represent correlations between genes (Wolfe et al., 2005). Significant edges can then be extracted using techniques such as random matrix theory (RMT) (Luo et al., 2007; Gibson et al., 2013) or soft thresholding as implemented in weighted gene co-expression network analysis (Langfelder and Horvath, 2008). Clustering techniques such as link community detection can be used to identify highly interconnected GCN subnetworks (modules) that are more likely to share common biological function or regulatory mechanisms (Ahn et al., 2010). Knowledge Independent Network Construction (KINC) is a software package that constructs GCNs with tracking of the samples used in edge detection. Prior to performing correlation analysis on a given gene pair, KINC identifies sample clusters using Gaussian mixture models (GMMs) (Ficklin et al., 2017). A correlation test (e.g. Spearman) is performed for each cluster separately, allowing significant GCN edges to be detected that are specific to a subset of the input samples. These edges are then annotated for attributes including genotype, phenotype, or experimental condition including time points. KINC is open source software and has been used successfully to detect condition-specific co-expression relationships in human data sets (Dunwoodie et al., 2018; Poehlman et al., 2019). In this study, we obtained RNA gene expression profiles from nodulating *M. truncatula* roots and combined differential gene expression analysis with a KINC GCN to identify sets of candidate nodulation genes.

## Methods

### Plant Growth Conditions, Inoculation, and Tissue Extraction

*M. truncatula* seeds were scarified for eight minutes using sulfuric acid, rinsed with water five times and imbibed in distilled water for 2 h. These seeds were cold treated at 4°C for 48 h in the dark in a moist environment (petri dish), followed by germination at room temperature for 18 h in the dark. The germinated seedlings were grown in an aeroponic apparatus and media as described previously (Penmetsa and Cook, 1997) following a 16 h/8 h light/dark cycle. At 3.5 h into the light cycle on the third day after loading onto the apparatus, a set of plants was marked with ink 1 cm from the root tip (at the distal end of the rhizobia-susceptible root maturation zone) to be used for tracking the location of the first developing nodules and 2 cm root sections starting 1 cm from the root tip were harvested from 10 experimental plants (0 h sample). *S. medicae* ABS7 (150 OD_600_ units) in caisson medium or bacteria-free caisson medium (mock inoculation) was then added to the apparatus. Tissue sections (2 cm) from the zone of developing nodules were harvested from 10 plants each at 12, 24, 48, and 72 h post-inoculation, using the marked plants to determine the location of the developing nodules. Three biological replicates of the time course for both inoculated and uninoculated roots were collected for use in RNA-Seq. A fourth replicate (repX) was performed under the same conditions for qRT-PCR confirmation.

### Ribonucleic Acid Extraction

Total RNA was isolated from *M. truncatula* root samples using the Invitrogen RNAqueous™ Total RNA Isolation Kit (Thermo Fisher, USA) according to the manufacturer’s instructions. The quality of RNA extracted was determined using a 2100 Bioanalyzer (Agilent, USA). All samples had an RNA Integrity Number (RIN) greater than 8.0. RNA samples were quantified using a Qubit Fluorometer (Thermo Fisher, USA).

### Transcriptome Data Generation

RNA-Seq libraries were made by the Clemson University Genomics and Computational Lab from 500 ng of total RNA using the TruSeq Stranded mRNA Library Prep Kit (Illumina, Cat. No. RS-122-2103) according to the manufacturer’s instructions. Samples included ERCC RNA Spike-In Mix 1 (Thermo Fisher, USA). Libraries were sequenced to a depth of at least 18,000,000 reads using paired end reads with an average read length of 125 bp at the David H. Murdock Research Institute (Charlotte, NC) on a HiSeq2500.

### Ribonucleic Acid Sequencing Data Processing

The PBS-GEM workflow [https://github.com/wpoehlm/PBS-GEM] was utilized to process RNA sequencing reads on Clemson University’s Palmetto Cluster. Poor quality sequences and adapters were removed using Trimmomatic-0.38 (Bolger et al., 2014). Cleaned reads were mapped to the Mt4.0v1 reference genome using hisat2-2.1.0 (Kim et al., 2015) with the following parameters: *hisat2 –rna-strandedness RF –min-intronlen 20 –maxintronlen 7000 -p 4 –downstream-transcriptome-assembly*. SAM alignment files were filtered to retain only unique primary alignments (MAPQ 60), sorted, and converted to BAM files using samtools-1.8 (Li et al., 2009). Reference gene abundances were estimated using stringtie-1.3.4d (Pertea et al., 2015; Pertea et al., 2016) with the following options: *stringtie –G –e –B –A*.

### Differential Gene Expression Analysis

Raw gene counts were calculated using the prepDE.py script that is provided with the StringTie Package [https://ccb.jhu.edu/software/stringtie/dl/prepDE.py]. Differential expression analysis was performed using the DESeq2 R package (Love et al., 2014), which internally normalizes for library size. Genes with total read counts of less than 50 were excluded from analysis. Control and inoculated samples were compared separately at each time point (0, 12, 24, 48, and 72 h) using the *DESeqDataSetFromMatrix* function with the following formula: *design = ~ condition*. Genes with an adjusted p value of less than 0.05 were considered to be significant.

### Gene Expression Matrix Preparation

Gene-level FPKM (fragments per kilobase of gene per million read pairs) were extracted from the gene abundance output files produced by StringTie and merged into a gene expression matrix (GEM) using a PBS-GEM Perl script. The matrix was log_2_ transformed and preprocessed using the preprocessCore R library (Tsygankov, 2018) to detect outliers and reduce technical noise. Pairwise Kolmogorov-Smirnov (KS) tests were performed to test for outlier samples (KS Dval > 0.15). No outlier samples were detected. The matrix was quantile normalized using the *normalize.quantiles* function. This normalized GEM was used to construct a GCN. Heatmaps and expression plots were generated using the *clustermap* and *tsplot* functions from the Seaborn Python package [https://seaborn.pydata.org/] which uses average-linked hierarchical clustering.

### Gene Co-Expression Network and Functional Enrichment Analysis

The OSG-KINC [https://github.com/feltus/OSG-KINC] (Poehlman et al., 2017) workflow was utilized to execute 10,000 KINC similarity jobs on the Open Science Grid with the following parameters: *kinc similarity–method pc –clustering mixmod –criterion ICL –min_obs 20*. Output was transferred to Clemson University’s Palmetto Cluster and decompressed. KINC threshold was executed with the following parameters: *kinc threshold –min_csize 20 –clustering mixmod –method pc –th_method pc –max_modes 5*. *A* significance threshold of 0.946100 was identified, and the GCN was extracted using the following KINC extract parameters: *kinc extract –clustering mixmod –method pc –th_method pc –th 0.946100 –max_modes 5*. Link community modules (LCM) were identified with the linkcomm R package (Kalinka and Tomancak, 2011), using the “single” hcmethod and a minimum cluster size of 3. Functional enrichment of LCMs was performed using the FUNC-E [https://github.com/SystemsGenetics/FUNC-E] script which performs a Fisher’s exact test similar to the DAVID method of enrichment analysis (Huang et al., 2007). Gene model annotations for the Mt4.0v1 genome were obtained from phytozome (Goodstein et al., 2012) and parsed for input into this script.

### Real Time Quantitative Polymerase Chain Reaction

For rep3 samples, RNA prepared for RNA-Seq from the third biological replicate was used. For repX samples, RNA was purified from an independent replicate of 2 cm root sections (as described above in RNA extraction) using the E.Z.N.A. Plant RNA Kit (Omega Bio-Tek; Norcross, GA). cDNA was synthesized from 300 ng RNA from each sample with the iScript cDNA Synthesis Kit (Bio-Rad; Hercules, CA) in a 20 µl reaction, subsequently diluted to 60 µl. Real Time qPCR was performed in 12.5 µl reactions in an iQ5 instrument (Bio-Rad, Hercules, CA) using iTaq™ Universal SYBR® Green Supermix (Bio-Rad, Hercules, CA), 0.35 µM of each primer (**Table S1**), and 2.5 µl of cDNA. Reactions were performed in three technical replicates and the average Ct calculated. Efficiencies (E) of each primer pair were determined from a dilution series of template and used to calculate relative expression. All primer pairs exhibited amplification efficiencies of greater than 1.9. Expression values for the genes of interest were calculated relative to expression of the housekeeping reference gene MtPI4K (Medtr3g091400) using the equation E_ref_^Ct_ref_/E_goi_^Ct_goi_.

## Results

### Network Creation

**Figure 1:**
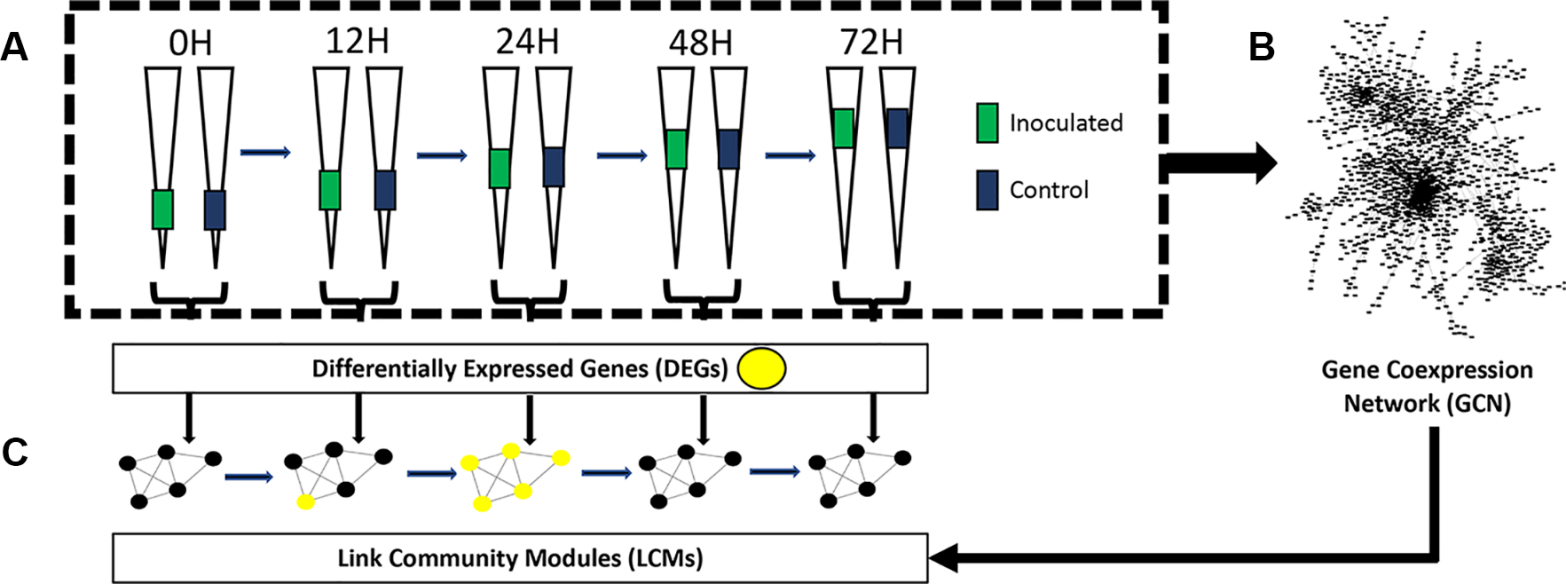
Experimental overview. **(A)** Differentially expressed genes between control and inoculated samples were identified at each time point. Elongated triangles represent roots; colored portions are the areas harvested (see *Methods*) for each comparison in the bracket at the bottom. **(B)** A gene co-expression network was constructed from all 30 samples (see *Methods*) and **(C)** link community modules were identified by Knowledge Independent Network Construction. Differentially expressed link community modules (LCMs) were then identified by overlaying differentially expressed genes (yellow dots in modules) from each time point onto the LCMs.

The aim of this study was to use KINC to identify multiple genes that demonstrate time point-specific expression patterns. To achieve this aim, we performed RNA-Seq on 30 maturation zone samples at 5 distinct time points: 0 h, 12 h, 24 h, 48 h, and 72 h post-inoculation or mock inoculation. At each time point, we analyzed three biological replicates of control samples (mock inoculation) and three biological replicates of samples that were inoculated by *Rhizobium*. We identified differentially expressed genes between control and inoculated samples at each time point and constructed a GCN from these samples. We identified LCM modules from this GCN and overlaid differentially expressed genes in order to identify modules that were differentially expressed at specific time points. An overview of the experimental workflow is presented in **Figure 1**.

**Figure 2:**
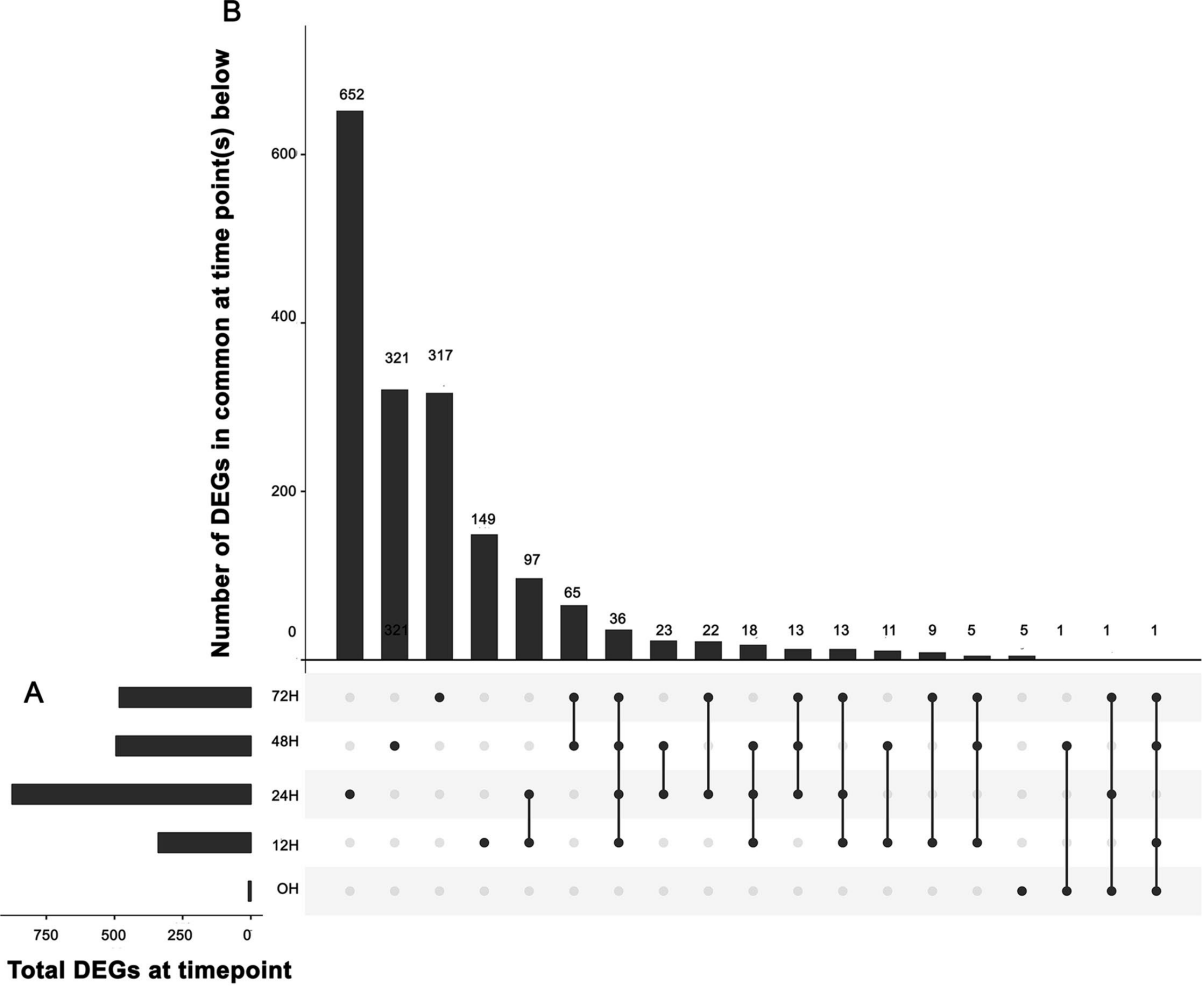
UpSet plot of differentially expressed genes. **(A)** Graph of total number of differentially expressed genes (DEGs) (X axis) at each time point (Y axis). **(B)** Intersection of sets of genes at multiple time points. Each column corresponds to a time point (first four columns) or set of time points (dots connected by lines below the X axis) containing the same DEGs. The number of genes in each set appears above the column, while the time points shared are indicated in the graphic below the column, with the time points on the left.

**Figure 3:**
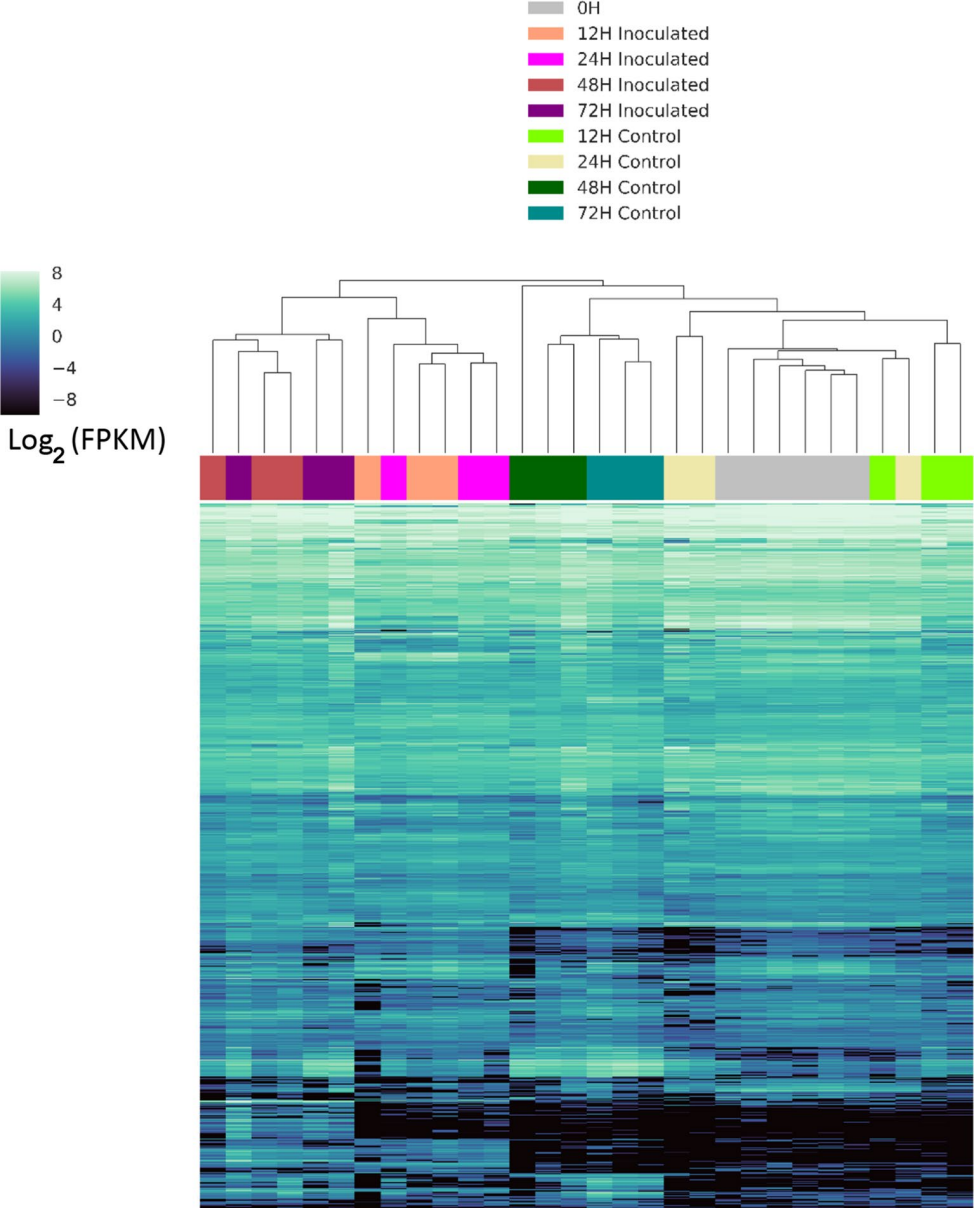
Clustered heatmap of differentially expressed genes. Expression is reported as Log_2_ of the fragments per kilobase of gene per million read pairs. Samples were clustered and visualized using the Seaborn clustermap function, which uses Euclidian distance metrics to generate a linkage matrix used for hierarchical clustering. X-axis across the top is the is dendrogram of samples at individual time points and conditions, indicated by the color key, while the genes are represented by individual lines on the Y axis as sorted by the clustermap function.

We identified genes that were differentially expressed between control and inoculated samples at each time point, resulting in a total of 1,758 differentially expressed genes (DEGs) at various time points (**Table S2**). An UpSet (Lex et al., 2014) plot in **Figure 2** shows that the majority of significant DEGs were specific to a single time point. However, we detected a core of 36 genes (arrow in **Figure 2**) that were differentially expressed from 12 h through 72 h (**Table S3**). We detected eight DEGs between the three samples used for 0 h inoculated and the three used for 0 h control, and five of these DEGS were unique to this time point. We detected 149 unique DEGs at 12 h, 652 unique DEGs at 24 h, 321 unique DEGs at 48 h, and 317 unique DEGs at 72 h (**Table S2**), unique meaning the gene is a DEG only at one time point. A heatmap of these clusters genes based on expression differences between control and inoculated samples (**Figure 3**). The dendogram on the X axis shows all control samples clustered together, mostly by time point, while the inoculated sample clusters tended to group by time point, with occasional blending of individual replicates at adjacent time points.

**Figure 4:**
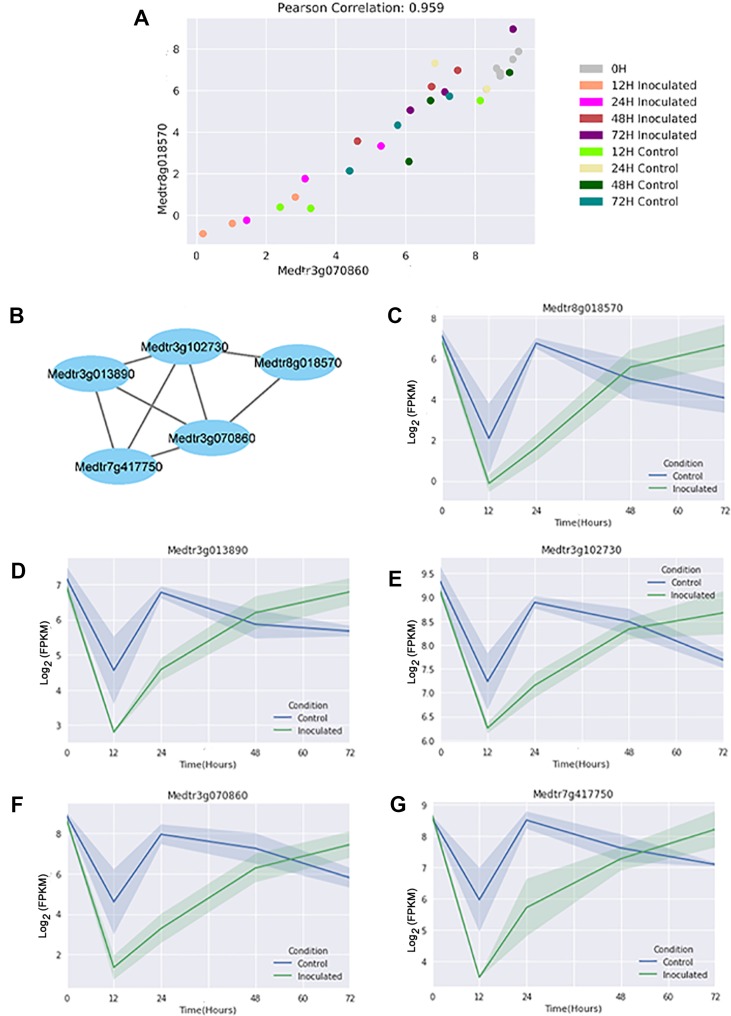
A representative gene co-expression network edge and link community module. **(A)** In this example, the Log_2_ fragments per kilobase of gene per million read pairs (FPKMs) of two genes selected from the module are plotted on the X and Y axis respectively. They show a high correlation value across all samples, both inoculated and control. **(B)** Relationships of the genes in the module. Edge length has no meaning beyond connection. **(C**–**G)** Expression plots (Log_2_ FPKMs *versus* time) for the individual genes in the module reveal that all genes have differential expression at the 24 h time point, but additionally share expression patterns at the other time points. Shading indicates 68% confidence interval of three independent replicates, and the point where genes are differentially expressed is marked-in this case where the shading does not overlap.

A normalized GEM constructed from all genes in these thirty samples was used to construct a GCN with KINC. The resulting GCN contains 4,067 nodes and 7,854 edges and showed scale-free topology (R2 = 0.799). **Figure 4** shows a representative GCN edge from two genes that are down regulated in inoculated samples at the 24 h time point. We observe that while the 24 h time point expression was used to select the genes, as expected, the overall pattern of expression in the time course is conserved among all genes in the module (**Figure 4**). We detected 161 LCMs that contained at least three genes, with the largest LCM containing 128 genes (**Table S4**).

### Functional Enrichment Analysis

We detected 53 unique DEGs that were present in LCMs. Nine of the LCMs detected were comprised entirely of genes that were differentially expressed at specific time points. We detected modules that were up-regulated at 24 h: M0004 and M0006. The genes in these modules are listed in **Table 1** and a heatmap of their expression patterns appears in **Figure S2A**. M0004 and M0006 are both enriched for Pfam (protein families database) terms PF01190 (“pollen proteins Ole e I like”) and PF09478 (“carbohydrate binding domain CBM4”) (**Table S5**). Conversely, we detected modules that were down regulated at 24 h: M0021, M0055, M0064, and M0072. The genes in these modules are listed in **Table 2** and a heatmap of their expression patterns appears in **Figure S2B**. M0021 is enriched for KEGG K13416 [“BAK1; brassinosteroid insensitive 1-associated receptor kinase 1 (EC:2.7.10.1 2.7.11.1)”]. M0055 is enriched for Pfam PF06351 (“allene oxide cyclase”). M0064 is enriched for GO:0008299 (“isoprenoid biosynthetic process”), GO:0004452 (“isopentenyl-diphosphate delta-isomerase activity”), K01823 [“idi, IDI; isopentenyl-diphosphate delta-isomerase (EC:5.3.3.2)”], K01597 [“MVD, mvaD; diphosphomevalonate decarboxylase (EC:4.1.1.33)”], K00787 [“FDPS; farnesyl diphosphate synthase (EC:2.5.1.1 2.5.1.10)”], PF00348 (“polyprenyl synthetase”), and PF00288 (“GHMP kinases N terminal domain”) (**Table S5**). We also detected modules that were down regulated at 48 h: M0032, M0118, and M0132. The genes in these modules are listed in **Table 3** and a heatmap of their expression patterns appears in **Figure S2C**. M0032 and M0132 are both enriched for K15401 [“CYP86A1; fatty acid omega-hydroxylase (EC:1.14.-.-)”]. M0132 is also enriched for PF04535 [“domain of unknown function (DUF588)”] (**Table S5**).

**Table 1:** Genes assigned to modules consisting of entirely of up regulated genes at 24 h post-inoculation (24U). If a gene appeared in multiple modules, only the last digit of the module number is listed for the additional modules. LogFC is the log_2_ fold change in expression between the two conditions for each gene and Padj is the Benjamin-Hochberg adjusted p value as reported by DESeq2.

Gene ID	Gene description	LCM module	LogFC	Padj
Medtr8g042900	Pectinesterase/pectinesterase inhibitor	M0004	3.23	4.17E−07
Medtr7g102770	Pollen Ole e I family allergens	M0004,6	2.82	1.23E−04
Medtr3g071470	Pollen Ole e I family allergens	M0004, 6	2.76	7.86E−04
Medtr4g074960	Endo-1,4-beta-glucanase	M0004	2.62	5.99E−03
Medtr2g035120	Disease-resistance response protein	M0004	1.73	8.41E−03
Medtr4g074960	Endo-1,4-beta-glucanase	M0006	2.62	5.99E−03
Medtr4g109880	Adenine nucleotide alpha hydrolase superfamily protein	M0006	1.98	1.33E−02

**Table 2:** Genes assigned to modules consisting entirely of down-regulated genes at 24 h post-inoculation (24D). If a gene appeared in multiple modules, only the last digits of the number are listed for the additional modules. LogFC is X, Padj is Y.

Gene ID	Gene description	LCM modules	LogFC	Padj
Medtr3g070860	Leucoanthocyanidin dioxygenase-like protein	M0021,55,72	−4.36	6.33E−05
Medtr2g008380	Somatic embryogenesis receptor-like kinase	M0021	−2.70	8.74E−05
Medtr3g013890	3-Oxo-delta(4,5)-steroid 5-beta-reductase-like protein	M0021,55	−2.28	4.85E−04
Medtr3g102730	3-Oxo-delta(4,5)-steroid 5-beta-reductase-like protein	M0021,55	−2.00	1.60E−03
Medtr8g018570	Seed linoleate 9S-lipoxygenase	M0055,72	−4.82	3.12E−08
Medtr7g417750	Allene oxide cyclase	M0055	−2.63	1.97E−02
Medtr1g112230	Mevalonate diphosphate decarboxylase	M0064	−2.49	4.97E−11
Medtr2g027300	Geranylgeranyl pyrophosphate synthase	M0064	−1.71	2.50E−20
Medtr7g080060	Isopentenyl-diphosphate delta-isomerase	M0064	−1.70	9.35E−04
Medtr7g085120	Nod factor-binding lectin-nucleotide phosphohydrolase	M0072	−3.82	1.37E−06
Medtr7g417750	Allene oxide cyclase	M0072	−2.63	1.97E−02

**Table 3:** Genes assigned to modules consisting entirely of down-regulated genes at 48 h post-inoculation (48D). If a gene appeared in multiple modules, only the last digits of the number are listed for the additional modules. LogFC is X, Padj is Y.

Gene ID	Gene description	LCM module	LogFC	Padj
Medtr5g014100	Anionic peroxidase swpb3 protein	M0032	−3.28	3.71E−05
Medtr2g062600	Lipid transfer protein	M0032, 132	−3.24	1.54E−06
Medtr8g089300	CASP POPTRDRAFT-like protein	M0032, 132	−2.88	4.25E−03
Medtr5g070010	Cytochrome P450 family-dependent fatty acid hydroxylase	M0032, 132	−2.87	1.72E−05
Medtr5g064530	Leguminosin group485 secreted peptide	M0118, 132	−3.13	1.75E−06
Medtr0097s0070	CASP POPTRDRAFT-like protein	M0118, 132	−3.04	4.96E−06
Medtr4g415290	Glycerol-3-phosphate acyltransferase	M0118, 132	−2.80	1.20E−05
Medtr1g071720	Lipid transfer protein	M0118	−2.46	4.90E−03
Medtr2g009450	Leguminosin group485 secreted peptide	M0132	−3.10	1.22E−05
Medtr8g079050	GDSL-like lipase/acylhydrolase	M0132	−3.00	2.61E−08
Medtr3g463060	Cytochrome P450 family-dependent fatty acid hydroxylase	M0132	−2.68	2.61E−06

**Figure 5:**
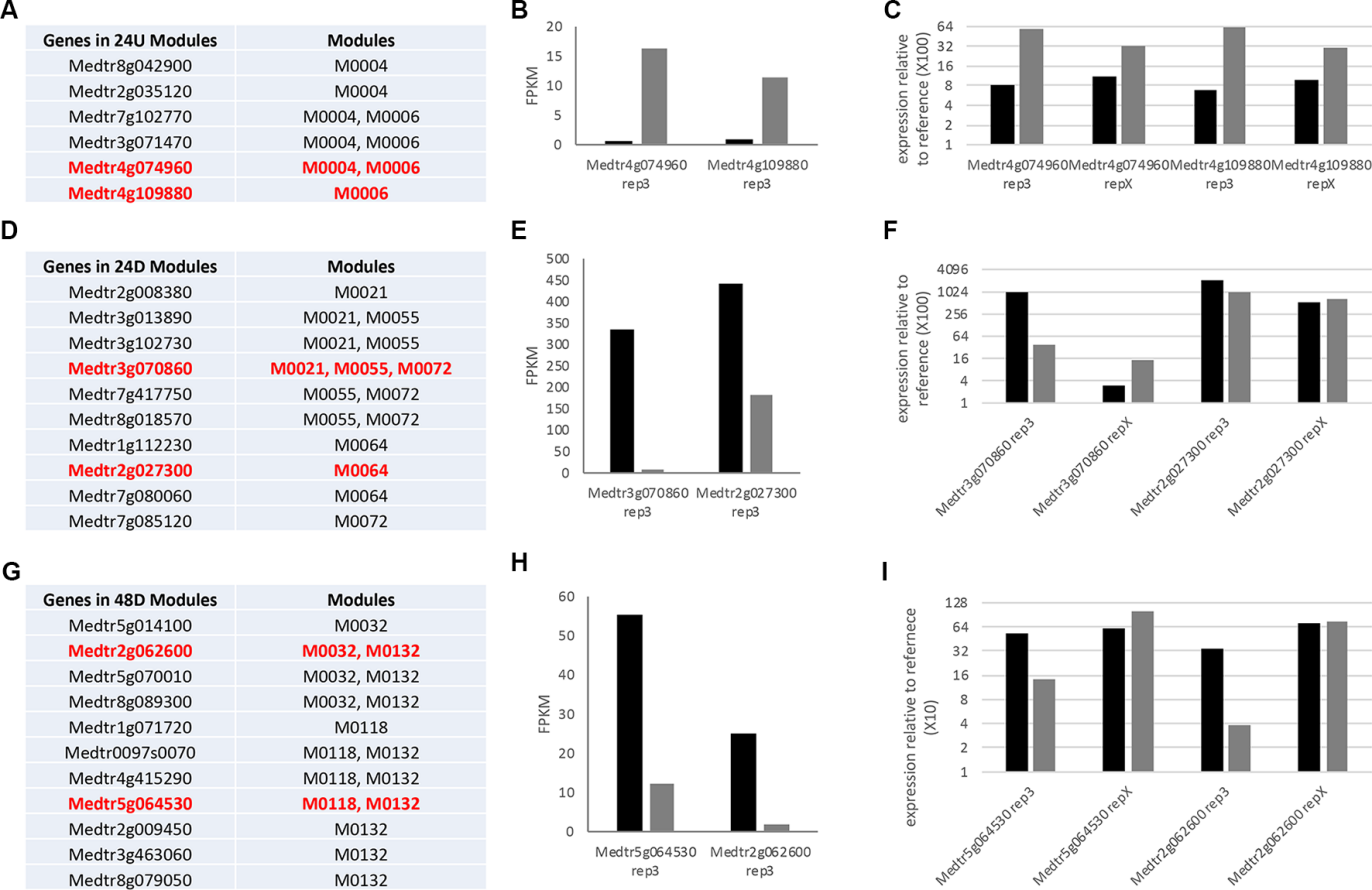
Investigation of module gene expression prediction. **(A**, **D**, **G)** List of genes contained in modules grouped in the text as composed completely of genes differentially expressed at one time point. Genes in red were used for further analysis here because they are not described in the text and they had similar expression levels aiding analysis. **(B**, **E**, **H)** Graph of the expression (fragments per kilobase of gene per million read pairs) of the genes in red in replicate 3 of the RNA-Seq. **(C**, **F**, **I)** Results of real time qRT-PCR analysis of expression of these genes in replicate 3 and an additional identical replicate x at the time point in question, displayed as relative expression to aid comparison. See *Methods* for primers and normalization calculation.

To determine if our LCM analysis could be extrapolated to other experiments, we first validated the expression of two genes selected from each of the collections of modules; the 24 h up-regulated modules in **Table 1** (24U), the 24 h downregulated modules in **Table 2** (24D), and the 48 h downregulated modules in **Table 3** (48D). Tested genes were chosen to cover as many modules as possible within an LCM. Using RNA from one of the three biological replicates used for RNA-Seq as the template for real time qRT-PCR (termed rep3), the expression pattern for all tested genes for the three modules was confirmed to be the same whether tested by RNASeq or real time qRT-PCR (**Figure 5**). We then tested the predictive power of the analysis by testing expression on RNA from an independent fourth biological replicate of the experiment (termed rep X) at the same time point by real time qRT-PCR (see *Methods*). Both genes from the 24U modules showed the expression pattern predicted from the modules (**Figure 5C**). The two tested genes from the 24D modules had the same expression pattern as each other, but the pattern at this one point in time differed from that identified in the initial experiment. (**Figure 5F**). Results from the two tested genes in the 48D modules were also inconclusive (**Figure 5I**) and we discuss this below.

## Discussion

We identified DEGs between control and inoculated samples at five distinct time points. With six biological replicates of the 0 time point, the identification of six DEGs from over 55,000 genes shows little biological variation in our method. As shown in **Figure 2**, the majority of these genes were unique to one specific time point, although those at two or more time points are of increased interest. Importantly, the set of 36 genes differentially expressed at all time points (**Table S3**) contain a large number of genes that have been identified by forward genetics as being important in the nodulation process (Ferguson et al., 2019). Finding new useful biological signals from hundreds of genes at each time point became a challenge, one we addressed by using the GCN to identify genes that display similar expression over the time series and comparing them to the DEG list at individual time points. **Figure 4** shows how two genes with a high correlation value in KINC did indeed have similar expression patterns over time, even though the genes were differentially expressed at the 24 h time point. We then identified LCMs from this GCN to find clusters of genes that all had similar expression patterns. **Figure 4** shows the relationships between the genes in LCM M055. Although it is comprised entirely of genes that are down regulated in inoculated samples at the 24 h time point, expression of these genes drops at the 12 h time point and then is restored at the 24 h time point in control samples, while the expression in the inoculated samples slowly rises for all genes (**Figures 4**). We detected 161 LCMs that displayed coordinated expression patterns and overlaid DEGs at each time point to these LCMs. We were able to detect nine LCMs that were composed entirely of genes that were either up or down regulated at a specific time point.

The two modules (M0004 and M0006) that are composed of up-regulated genes at 24 h are enriched for Pfam term PF01190 (“pollen proteins Ole e I like”). Pollen allergen genes have undergone a high degree of duplication and purifying selection, suggesting that they are maintained because of unique biological functions (Chen et al., 2016). Some of these functions include defense response to bacterium and cell redox homeostasis, two processes that are involved in root nodulation, suggesting there may be additional functions for these genes in *M. truncatula*. The genes in **Table 1** are also enriched for PF09478 (“carbohydrate binding domain CBM49”), a group of cellulases often associated with cell wall hydrolysis (Urbanowicz et al., 2007). Notably, **Table 1** contains a pectinesterase gene (Medtr8g042900) and a disease response gene (Medtr2g035120). Thus, the up-regulated genes in **Table 1** could be involved in pathogen response or cell wall remodeling, important aspects of infection thread penetration, and nodule development. Aspects of a pathogen response, such as ROS production, occurs upon rhizobial inflection and then are quickly tamped down during infection, and the timing of this is consistent with that process (Plett and Martin, 2017). The two genes tested on a fourth biological replicate are independent from genes described above and in this additional replicate they showed the same pattern of regulation as the other three replicates, suggesting the pattern is consistent, but firm conclusions cannot be drawn without further testing.

**Table 2** contains GCN modules that are down regulated in inoculated samples at 24 h. M0072 and M0055 both contain a gene related to jasmonic acid (JA) synthesis: Medtr7g417750 (allene oxide cyclase). Suppression of this gene has been shown to reduce JA levels in hairy roots of *M. truncatula*, lowering the plant’s ability to achieve mycorrhization (Isayenkov et al., 2005). While JA seems to play a positive role in mycorrhization, it has been demonstrated to negatively impact root nodulation by inhibiting nod-factor induced calcium oscillations in the nucleus of the cells (Sun et al., 2006). Interestingly, JA and cytokinin were found to have antagonistic roles in *Arabidopsis* xylems (Jang et al., 2017). We speculate that down-regulation of genes in **Table 2** results in a decrease in JA production and an increase in cytokinin biosynthesis, contributing to root nodulation by shutting down alternate pathways that would otherwise enable mycorrhizal symbiosis. We found Medtr7g085120, a Nod factor-binding lectin-nucleotide phosphohydrolase, to be down-regulated in inoculated samples at this time point. This protein is necessary for rhizobial and mycorrhizal symbiosis in *Lotus japonicus*, a determinate nodulating plant (Roberts et al., 2013). Previous studies that analyzed RNA expression levels of whole-root tissue found this gene to be up-regulated early in the course of *Sinorhizobium meliloti* response in *M. truncatula*. We speculate that the cellular composition of the tissue used in our study demonstrates differential expression of this gene compared to the whole-root samples previously analyzed (Larrainzar et al., 2015). The two genes tested on a fourth biological replicate X are not any of the genes described above. In replicate X, both genes from this module showed the same pattern of regulation as each other, but they both appeared to be slightly upregulated in this single biological replicate. Because this is a single replicate, no conclusion can be drawn, but if there is a difference it suggests there may be another environmental factor beyond nodulation that leads to their co-regulation (one gene appears in multiple modules) and that factor is different in replicate X. More likely, since expression of the two genes tested is flat in response to rhizobia 24 h in the RNASeq but up by 72 h (**Figure S2B**) it is possible that the degree of downregulation and the overall trend are important to prediction from the modules.

**Table 3** contains two modules, M0032 and M0132, that are enriched for KEGG orthology term K15401 (“fatty acid omega-hydroxylase”). All three modules (M0032, M0132, and M0118) contain genes that are annotated as “lipid transfer protein”. Lipids play diverse roles in plant physiology, such as signaling pathways involved in plant defense (Pinot and Beisson, 2011; Waschburger et al., 2018). Notably, Medtr4g415290—a glycerol-3-phosphate acyltransferase (GPAT) gene, is down-regulated in both M0132 and M0118. GPAT enzymes catalyze the first step of membrane phospholipid biosynthesis (Takeuchi and Reue, 2009; Waschburger et al., 2018). Another GPAT gene in *M. truncatula*, RAM2, is necessary for fungal mycorrhization through its involvement in cutin biosynthesis (Wang et al., 2012). Other genes involved in lipid biosynthesis are present in **Table 3**: Medtr5g070010 (“cytochrome P450 family-dependent fatty acid hydroxylase”), Medtr8g079050 (“GDSL-like lipase/acylhydrolase”), and Medtr3g463060 (“cytochrome P450 family-dependent fatty acid hydroxylase”). We hypothesize that down-regulation of genes in **Table 3** results in inhibition of synthesis of specific fatty acids that would otherwise play a negative role in root nodulation. M0032 also contains a peroxidase protein, Medtr5g014100. Given that peroxidases are often involved in stimulating plant defense against pathogens (Almagro et al., 2009), we hypothesize that down-regulation of this gene helps to enable rhizobial infection. In the additional biological replicate X, the two genes tested, which we not in the group discussed above, did not exhibit strong down regulation in the single replicate but rather the relative expression differences between the two genes were close. Again, because this is a single biological replicate no firm conclusion can be drawn, but since this is also a downregulated module in which all the genes are upregulated at 72 h (**Figure S2C**), it could be seen as further confirmation that the degree of downregulation and the overall trend are important to prediction from the modules. Especially because both genes tested appear in more than one module, we also cannot eliminate that there may be another environmental factor beyond nodulation that leads to the co-regulation and that factor is different in replicate x.

Many of the genes in **Tables 1**–3 are involved in pathogen response. Given that the genes in **Table 1** are up-regulated in inoculated samples, these genes might play a role in normal pathogen response, while the down-regulated genes in **Tables 2** and **3** could play important roles in damping response during nodulation. To support this, we compared the genes in these tables to genes that have been found to be dysregulated in *nad1* mutants. *NAD1* (nodules with activated defense 1) is a gene necessary for maintaining rhizobial symbiosis in *M. truncatula* roots (Wang et al., 2016; Domonkos et al., 2017; Yu et al., 2018). In *nad1* mutants, brown pigmentation accumulates in the nodules following the release of *Rhizobium* from the infection thread, resulting in nodule necrosis. Wang et al. performed transcriptome profiling of *nad1* mutants to compare with control plants at 21 days post-inoculation (Wang et al., 2016). Out of the six total genes we identified in **Table 1**, three were upregulated in *nad1* mutants (Medtr3g071470, Medtr4g109880, Medtr7g102770). Out of the 10 genes identified in **Table 2**, 5 were up-regulated in *nad1* mutants (Medtr8g018570, Medtr3g070860, Medtr7g417750, Medtr3g102730, Medtr3g013890), while 1 gene (Medtr7g085120) was down-regulated in the mutants. Six of the 11 genes in **Table 3** were up-regulated in the mutants (Medtr0097s0070, Medtr3g463060, Medtr5g070010, Medtr8g079050, Medtr4g415290, Medtr5g064530), while one gene was down-regulated (Medtr5g014100). Given that NAD1 plays a key role in regulating immune response to *Rhizobium*, genes that are dysregulated in NAD1 mutants may play key roles in nodulation (Wang et al., 2016). Thus, we speculate that the down regulation of genes in **Tables 2** and **3** helps to suppress innate immune responses that would otherwise prevent rhizobial colonization in nodules.

The differentially expressed LCMs we identified provide information about coordinated regulation, with the caveat that additional biological testing should be used to confirm LCM members with downregulation. The tested co-regulated genes identified as downregulated suggest the downregulation prediction may not be robust for extrapolation with this software. Further research is needed to determine if the expression patterns of these genes are causative biomarkers, or if they are simply an effect of root nodulation or pathogen defense pathways, but their identification suggests hypotheses for testing. Regardless, these LCMs revealed biochemical differences between control and inoculated samples over the course of root infection. This study also provides a list of DEGs from the maturation zone of *M. truncatula* roots for further analysis. While this investigation focused on the LCMs that were composed only of genes that were differentially expressed, other LCMs in which a subset of the genes were differentially expressed are the subject of continued investigation in our lab. Our work describes a framework for creating networks that will be investigated in future wet and dry lab experiments.

## Data Availability Statement

The raw FastQ files generated for this study can be downloaded from the NCBI SRA database [https://www.ncbi.nlm.nih.gov/sra] under BioProject PRJNA524899 (SRA accessions: SRR8650758-SRR8650787).

## Author Contributions

FF and JF conceived the study. ES and SC grew plants and extracted RNA. ES performed qRT-PCR analysis. WP, ES, and FF performed data analysis. WP, ES, FF, and JF wrote the manuscript. All authors have read and approved the manuscript.

## Funding

This work was supported by the National Science Foundation Awards #1659300 and #1444461.

## Conflict of Interest

The authors declare that the research was conducted in the absence of any commercial or financial relationships that could be construed as a potential conflict of interest.
