# Supplementary figures and images for: Identifying Temporally Regulated Root Nodulation Biomarkers Using Time Series Gene Co-Expression Network Analysis
Source: Front Plant Sci. 2019 Oct 31;10:1409. doi: 10.3389/fpls.2019.01409 (PMC6836625; doi:10.3389/fpls.2019.01409)

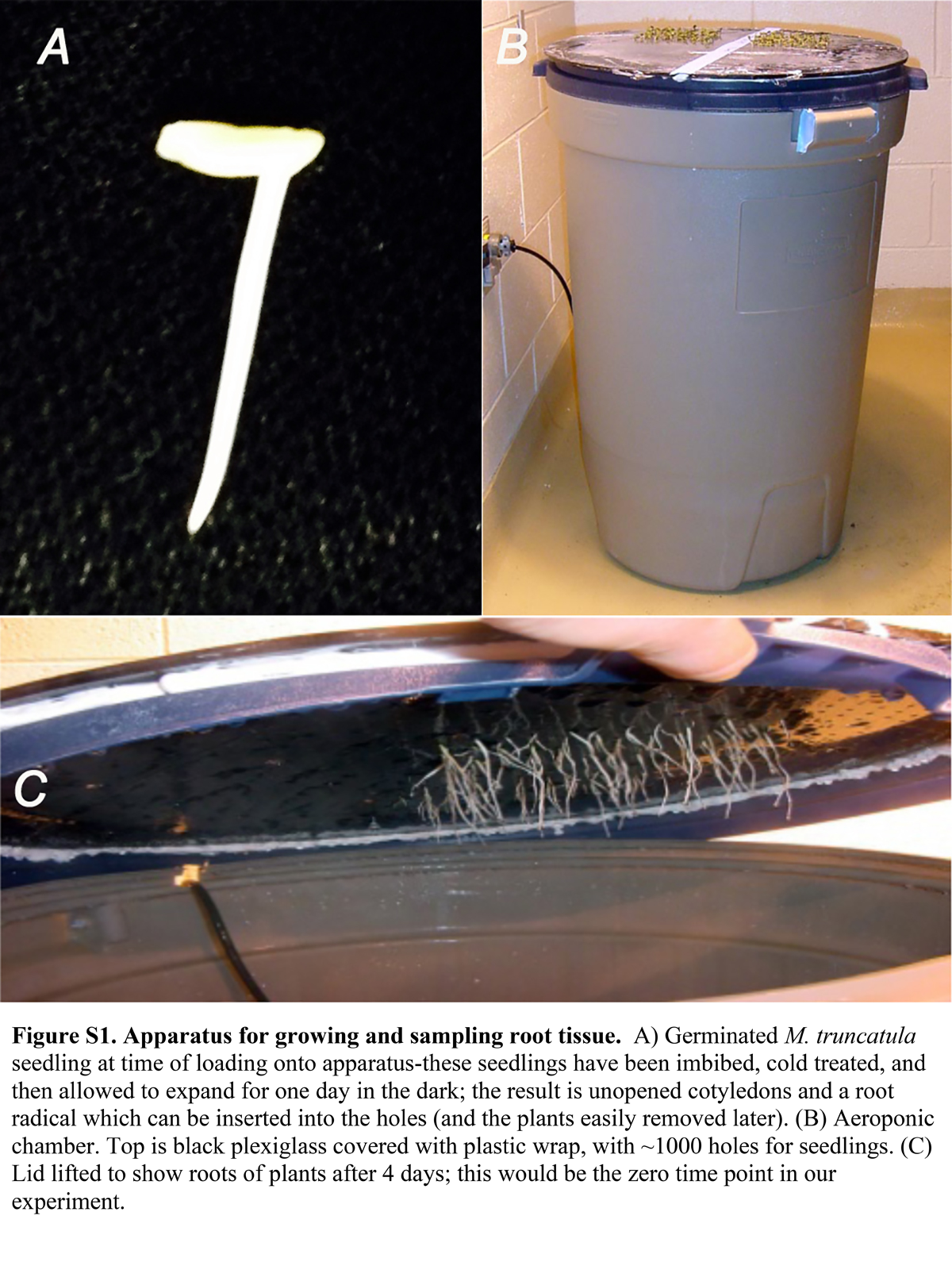

Supplement: Supplementary file 1 [file Image_1.tif]

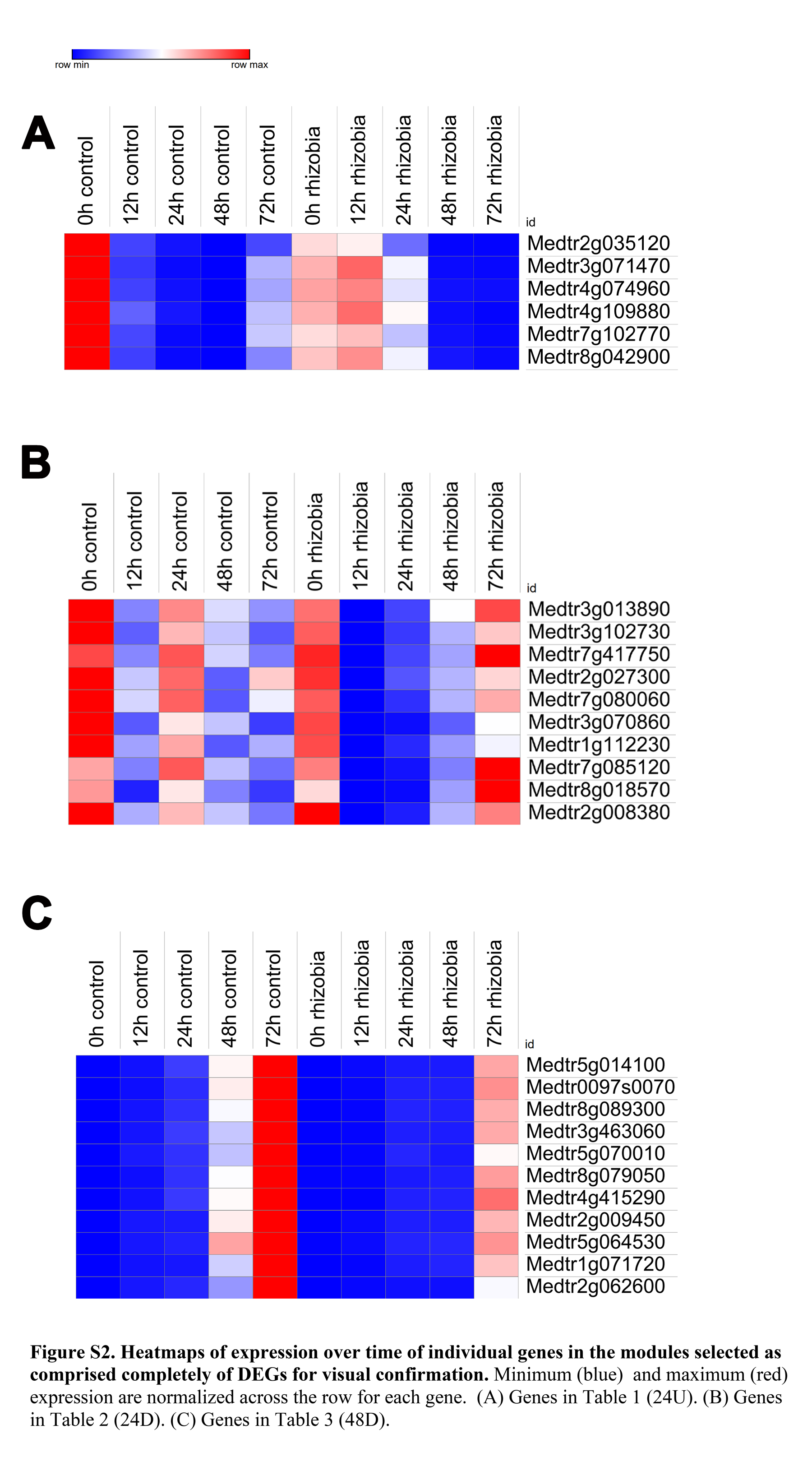

Supplement: Supplementary file 2 [file Image_2.tif]
